# Multilevel latent class casemix modelling: a novel approach to accommodate patient casemix

**DOI:** 10.1186/1472-6963-11-53

**Published:** 2011-03-01

**Authors:** Mark S Gilthorpe, Wendy J Harrison, Amy Downing, David Forman, Robert M West

**Affiliations:** 1 Centre for Epidemiology & Biostatistics, School of Medicine, University of Leeds, Worsley Building, Clarendon Way, Leeds, LS2 9JT, UK; 2 School of Healthcare, University of Leeds, Baines Wing, University Campus, Leeds, LS2 9UT, UK; 3 Leeds Institute of Health Sciences, School of Medicine, University of Leeds, Charles Thackrah Building, Clarendon Road, Leeds, LS2 9LJ, UK; 4 Northern and Yorkshire Cancer Registry and Information Service, Level 6 Bexley Wing, St James's Institute of Oncology, Leeds, LS9 7TF, UK; 5 Cancer Information Section, International Agency for Research on Cancer, 150 cours Albert Thomas, F-69372 Lyon Cedex 08, France

## Abstract

**Background:**

Using routinely collected patient data we explore the utility of multilevel latent class (MLLC) models to adjust for patient casemix and rank Trust performance. We contrast this with ranks derived from Trust standardised mortality ratios (SMRs).

**Methods:**

Patients with colorectal cancer diagnosed between 1998 and 2004 and resident in Northern and Yorkshire regions were identified from the cancer registry database (n = 24,640). Patient age, sex, stage-at-diagnosis (Dukes), and Trust of diagnosis/treatment were extracted. Socioeconomic background was derived using the Townsend Index. Outcome was survival at 3 years after diagnosis. MLLC-modelled and SMR-generated Trust ranks were compared.

**Results:**

Patients were assigned to two classes of similar size: one with reasonable prognosis (63.0% died within 3 years), and one with better prognosis (39.3% died within 3 years). In patient class one, all patients diagnosed at stage B or C died within 3 years; in patient class two, all patients diagnosed at stage A, B or C survived. Trusts were assigned two classes with 51.3% and 53.2% of patients respectively dying within 3 years. Differences in the ranked Trust performance between the MLLC model and SMRs were all within estimated 95% CIs.

**Conclusions:**

A novel approach to casemix adjustment is illustrated, ranking Trust performance whilst facilitating the evaluation of factors associated with the patient journey (e.g. treatments) and factors associated with the processes of healthcare delivery (e.g. delays). Further research can demonstrate the value of modelling patient pathways and evaluating healthcare processes across provider institutions.

## Background

Survival from cancer varies according to many factors including place of diagnosis and treatment centre (Trust), [1,2] stage at diagnosis, [3,4] and associated risk factors such as age at diagnosis, sex, and socioeconomic background (SEB) [5-9]. Some Trusts perform better or worse than others in terms of average survival rates perhaps due to patient casemix at the time of entry into the healthcare system, though patient outcome differences will reflect underlying differences in the effectiveness of healthcare organisations. Much interest lies in identifying good and poor performing healthcare providers, to identify best practice and advocate changes in under-performing institutions. It is important to account for patient casemix when evaluating institutional performance and there are currently several strategies.

Regression (linear or logistic) is a traditional and well-documented approach, [10] where variables relating to patient characteristics are modelled, effectively to adjust the outcome in relation to the likely influences of these factors. Methods such as matching, stratification, [10] or propensity score analysis, [11,12] may also be used, though these techniques make potentially untestable assumptions and never account for the impact of unmeasured variables or accommodate Trust-level variation. Although multilevel modelling accounts for patients nested within Trusts, and provides improved estimates compared with logistic regression, [13,14] parametric assumptions are made that may not be tenable. Other methods, such as boosted decision trees, [15] have occasionally been used, though these can be difficult to interpret.

No casemix-adjustment strategy will eliminate all bias due to unmeasured differences amongst patients; [16] some procedures increase bias [17]. Accommodating patient variation through measured variables only is crude: models ought to reflect the uncertainty associated with patient casemix characteristics. Furthermore, casemix adjustment does not account for differences in patient treatments. Failure to capture variation in patient pathways and their consequences may result in over-simplistic interpretation of healthcare processes and consequent outcomes. Models need to accommodate patient casemix, the patient experience, and uncertainty in both.

Multilevel latent class (MLLC) modelling is proposed to: (i) adjust for patient casemix whilst accommodating uncertainty surrounding unrecorded patient characteristics; (ii) adjust for patient pathways in terms of the delivery of appropriate healthcare (e.g. treatments); and (iii) differentiate patient outcomes in relation to institutional process characteristics (e.g. delays to treatment). To demonstrate and validate all three steps simultaneously is challenging. The first of these is explored here. We contrast the MLLC model ranking of Trust performance with that of ranks derived from calculating Trust standardised mortality ratios (SMRs). To illustrate our methodology, we study routine data on colorectal cancer patients from a large UK health region.

## Methods

### The illustrative colorectal cancer dataset

Patients with colorectal cancer (ICD10 [18] codes C18, C19 and C20) diagnosed between 1998 and 2004 and resident in the Northern and Yorkshire regions were identified from the Northern and Yorkshire Cancer Registry and Information Service (NYCRIS) database. Patient age, sex, tumour stage at diagnosis (using the Dukes classification [19]), Trust of diagnosis/treatment, and whether or not the patient received treatment were extracted. Initial data extraction yielded 26,455 unique patient records. Socioeconomic background (SEB) was defined at the 2001 enumeration district level of residence (super output area) using the Townsend Index [20] and matched to patients using postcode. The primary outcome was dead or alive three years following diagnosis, which is clinically meaningful since colorectal cancer has a median survival of approximately three years and survival to three years is often considered for policy reasons.

An area deprivation score could not be obtained for one case. Patients with age at diagnosis greater than 100 years (7 patients) and patients identified by death certificate only (364; 1.4%) were excluded. Some patients had multiple diagnosis codes and for patients attending more than one hospital (16,549; 63%), the location of the most recent Trust with a relevant diagnosis code was recorded as the diagnostic/treatment centre, as this provided the latest staging information. For patients who did not have a relevant diagnosis code for any Trust visits (220; 0.83%), the location of their first Trust visit was taken as the diagnostic/treatment centre. Some 1,239 (4.7%) patients were excluded as their diagnostic centres were outside the NYCRIS region. Following exclusions, 24,640 (93%) of the identified patients remained for analysis.

### Statistical methods

Latent class analysis (LCA) is well established within single-level regression analysis. Also known as *discrete latent variable modelling*, or *mixture modelling*, one determines a number of latent classes, or subgroups, the optimum choice of which is typically informed by log-likelihood statistics. The Bayesian Information Criterion (BIC), [21] the Akaike Information Criterion (AIC), [22] and changes in log-likelihood (LL) are used as model-fit indicators, though models might also be selected on the basis of interpretation [23]. Model parameters of each latent class are determined empirically, along with their contribution to the outcome distribution. LCA models are useful where subtypes are sought and one wishes to model uncertainty surrounding class membership, since observations may belong to all classes, with probabilities determined empirically. LCA thus reflects the uncertainty associated with a limited number of predictors when determining subtypes of outcomes. The proposed LCA models are multilevel because patients are nested within diagnostic/treatment centres (Trusts). LCA extends to a multilevel setting by incorporating discrete latent variables at all levels of the hierarchy. For the colorectal cancer data, latent classes at the patient level model uncertainty surrounding affiliation to patient subgroups and latent classes at the Trust level model Trust variation. The modelling strategy was to determine patient-level latent classes (having included patient-level covariates) with Trust-level variation accommodated initially by a *continuous *latent variable. With patient-level subtype structure fixed, Trust classes were then sought by switching the Trust-level latent variable from *continuous *to *categorical*. A minimum of two Trust classes was required to exhibit discretised Trust class differences in patient outcomes.

The proposed modelling strategy builds upon work originated by Downing *et al*., [24] where multilevel LCA circumnavigated potential bias due to the 'reversal paradox' when adjusting for confounders on the causal path between exposure and outcome [25]. We have no such concerns here, since we are not seeking *inference *of any exposure nor confounder adjustment: rather, we seek to optimise outcome *prediction *by modelling patient characteristics to accommodate casemix differences. Consequently, all available covariates for which there was complete data (*age*, *sex*, and *SEB*) were considered by the modelling process, along with *stage *at diagnosis (coded A to D for increasing severity and missing coded X). Stage was included despite a degree of missing data (13.1%), because it is known to influence survival, [3,4] and a missing category was conveniently added. Although additional patient variables were available, such as time-to-first-treatment and treatment-received, these had substantial incomplete data that would question their utility and were therefore not used. Patient *age *at diagnosis and Townsend score (*SEB*) were continuous measures; *age *was centred on the study mean (71.5 years) and *SEB *was centred on the population mean of zero (study mean was -0.040). Both covariates exhibited a non-linear relationship with 3-year survival, so a quadratic term for *age *was included in the model; and by 'trimming' the tails of *SEB *(assigning rare values > ± 5.0 as ± 5.0), it was possible to avoid higher order terms for Townsend score. The model is described in the Appendix.

SMRs were calculated for each Trust (standardised by age, sex, deprivation and stage) and a scaled difference from 'SMR = 1' was determined for each Trust by dividing by the square root of the Trust size. For both the SMRs and the MLLC models, 200 bootstrapped datasets were generated and each was analysed in the same manner to determine 95% confidence intervals (CIs). We used MLLC to calculate absolute differences in Trust effects on the log odds scale (with patient-level values aggregated to the Trust level) before ranking in order of 'best' to 'worst' survival, to compare with the ranks generated from the Trust SMRs. For data manipulation, summary statistics, tabulation, and charts, Stata was used; [26] for latent variable models, LatentGold [27] was used.

## Results

Table 1 summarises the 'ideal' MLLC model determined by the procedures described. Patients were assigned to two latent classes of similar size, one with reasonable prognosis (PC1: 54.3% of cases, of which 63.0% died within three years), and one with better prognosis (PC2: 45.7% of cases, of which 39.3% died within three years). Trusts were similarly assigned to two latent classes. The largest Trust class, with 53.1% of patients, had better prognosis (TC1: 51.3% of patients died within three years; TC2: 53.2% of patients died within three years). Table 2 summarises the number of deaths within each patient class by stage. Allocating patients to classes according to their largest class probability (modal assignment), all patients in PC1 diagnosed either at stage B or C died within three years; in PC2, all patients diagnosed at stage A, B or C survived. This difference is anticipated, as stage at diagnosis is an important predictor of survival. Most of the early- or mid-stage patients died within three years in PC1 compared to PC2, and there was a clear graduation in survival with increasing stage at diagnosis from early- to late-stage within both classes. The predictor age differed substantially across classes. In contrast, the predictors deprivation and sex differed only marginally between patient classes.

**Table 1:** Results for the subject classes in the 2-patient, 2-Trust-class multilevel latent class regression model

Model Summary Statistics	Class 1	Class 2
Class Size	54.3%	45.7%
Overall Prevalence	63.0%	39.3%
Reference Group Prevalence	23.2%	7.0%

**Model Covariates**	**Odds Ratio (95% CI)**	

Stage = B	2.40 (1.63-3.54)	0.55 (0.21-1.43)
Stage = C	7.72 (4.61-12.94)	1.74 (0.75-4.06)
Stage = D	20.19 (8.88-45.89)	Infinite^†^
Stage = X	6.30 (1.89-20.97)	33.41 (7.93-140.68)
Female	0.94 (0.78-1.14)	0.58 (0.38-0.88)
Townsend (per SD more)	1.32 (1.21-1.43)	1.03 (0.81-1.31)
Age (per 5 years older)	1.51 (1.42-1.60)	2.53 (1.31-4.90)
Age squared (per 5 years older)	1.005 (0.997-1.012)	0.984 (0.960-1.008)

Odds ratio of death within 3 years. CI: Confidence Interval. There were 12,856 (52.2%) deaths in the study population. The reference group comprised males, aged 71.5 years, classified as Stage A at diagnosis, and attributed a Townsend score of zero. ^†^The odds ratio could not be estimated as there were zero patients who survived 3 years in this subcategory.

**Table 2:** Deaths by stage, and patient class, for the 2-patient, 2-Trust multilevel latent class regression model

	Modal Class			
	
	1		2	
Stage at Diagnosis				
	Died within 3 years		Died within 3 years	
	
	No	Yes	No	Yes
A	1099	550	1210	0
B	0	1955	4829	0
C	0	2736	3437	0
D	437	3202	0	1962
X	413	2360	359	91

TOTAL	1949	10803	9835	2053

**Figure 1:**
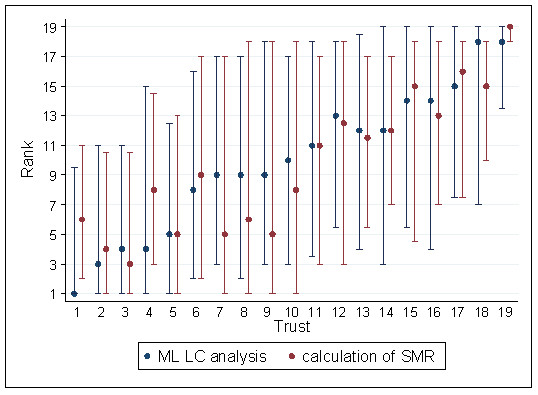
**Trust Median Ranks and 95% Confidence Intervals, ordered by the MMLC analysis**.

Trust ranks and their bootstrapped 95% CIs are summarised in Table 3; a low ranking value indicates a better survival rate than expected. Differences in the median rank of Trust performance between the MLLC model approach and the Trust SMRs are within their estimated 95% CIs. Figure 1 provides a graphical representation of these results, in order of increasing median probability of belonging to the best survival Trust class by the MLLC methodology.

**Table 3:** Trust ranks from the MMLC model and the calculation of Trust SMRs

Trust	Median probability of belonging to best survival Trust class	Median Rank (95% CI)	
		
		ML LC	SMR
1	1.000	1 (1-9.5)	6 (2-11)
2	0.999	3 (1-11)	4 (1-10.5)
3	0.997	4 (1-11)	3 (1-10.5)
4	0.996	4 (1-15)	8 (3-14.5)
5	0.993	5 (1-12.5)	5 (1-13)
6	0.956	8 (2-16)	9 (2-17)
7	0.912	9 (3-17)	5 (1-17)
8	0.908	9 (2-17)	6 (1-18)
9	0.897	9 (3-18)	5 (1-18)
10	0.816	10 (3-17)	8 (1-18)
11	0.575	11 (3.5-18)	11 (3-17)
12	0.476	13 (5.5-18)	12.5 (3-18)
13	0.372	12 (4-18.5)	11.5 (5.5-17)
14	0.359	12 (3-19)	12 (7-17)
15	0.152	14 (5.5-19)	15 (4.5-18)
16	0.070	14 (4-19)	13 (7-18)
17	0.070	15 (7.5-19)	16 (7.5-18)
18	0.003	18 (7-19)	15 (10-18)
19	0.002	18 (13.5-19)	19 (18-19)

## Discussion

In a standard multilevel setting, where a continuous latent variable is adopted at the Trust level, the implicit assumption is that Trust-level outcomes have an underlying normal distribution (conditional on Trust-level covariates): Trusts are effectively treated as a random sample of a larger (infinite) population of Trusts. Trusts are not, however, randomly placed geographically and nor are patients randomly assigned to Trusts. Parametric assumptions were therefore replaced by other assumptions which are less restrictive by adopting discrete latent variables, although there remains a degree of geographical dependency that is not accounted for. This remains a limitation. The simplest MLLC model adopted was therefore where the continuous latent variable at the upper level is replaced by a categorical latent variable. The model estimates the mean outcome for each Trust class and the size of each Trust class (summation of Trust probabilities for each Trust class) and no assumptions were made regarding the underlying distribution or class sizes. More complex models can extend this approach to accommodate the spatial dependencies, though this will be part of future developments.

An upper-level discrete latent variable allows for individual Trusts to be assigned probabilistically across the discrete latent classes, providing less restricted weighting of Trust relative performance. This may improve the accuracy of the estimated patient outcome differences across Trust classes, which improves the estimated patient casemix adjustment for individual Trusts. The MLLC model is more likely to capture contextual effects due to the inherent data hierarchy than either a standard multilevel approach or by merely estimating Trust ranks according to their SMRs. Continuous and discrete latent variables, if combined, may prove more parsimonious, with variation within each Trust class captured by the continuous latent variable, potentially leading to fewer Trust classes needed to describe overall Trust-level variation. Where determination of Trust ranks is important, the estimation of Trust outcomes is simpler if the categorical latent variable only is adopted at the Trust level, avoiding derivation of the normally distributed effects within each Trust class. Addressing spatial dependencies amongst the Trusts may nevertheless warrant incorporating upper-level effects.

In fixing patient-level latent class composition and accommodating patient casemix differences, the residual Trust-class differences in outcome reflect variations in Trust performance that depend upon Trust characteristics (differences in the treatments given *and *healthcare delivery processes). Model improvement might be feasible with more patient-level variables, but this would incorporate incomplete data, which can cause bias. Within a latent class framework the uncertainty surrounding unrecorded or unused patient characteristics is modelled explicitly: 'fuzzy' matching. Trust-level covariates might explain some of the Trust-class outcome differences if included. The optimum number and composition of Trust (and possibly patient) classes may change with the inclusion/exclusion of different covariates.

The probabilities of Trust class membership in Table 3 were marked, with most Trusts belonging entirely or predominantly to one Trust class. This is unsurprising, as there is only a modest difference between the two classes in median survival, and probabilistic assignment differentiates between the two, providing a class weighted combined survival rate. It is not feasible, however, for a Trust to be assigned a class weighted survival rate below that of the poorer survival class, or above that of the better survival class. This is an implicit constraint on the estimated weighted survival for Trusts allocated entirely to one of the two classes (e.g. Trust 1). To alleviate this, more Trust-level classes could be sought, increasing the number until no Trust had a probabilistic assignment of exactly one for classes at the extremities of the range of Trust outcome means. More research is needed, but as applied here, the estimated ranks are robust.

Although the analyses undertaken were primarily for illustration of the proposed methodology, the results are to be taken seriously. Bias may have occurred, however, due to patients with more than one Trust visit having been assigned the most recent Trust visited as the treatment centre. If diagnosis was made at a separate Trust to that which subsequently provided treatment, it would be the latter that was important when modelling healthcare delivery and process variables. In our dataset, 75% of patients visited only one Trust. Nevertheless, some inaccuracies may remain, which could be addressed by screening each patient journey to determine where the majority of interventions take place, or by using multilevel multiple membership models for multiple treatment centres. Furthermore, technically, we have cross-classified data, with patients nested in both area of residence (which yields the patient SEB) and diagnostic centre (Trust); the area level is thus crossed with the Trust level. The number of patients in each area, however, is small and for simplicity of illustration we discarded this level in our model. The methodological principles of MLLC modelling extend theoretically to a cross-classified context, but software does not yet facilitate this.

We have satisfactorily demonstrated the principles of step (i) outlined previously, but there is more research to be undertaken to determine the processes for steps (ii) and (iii), which embark upon modelling patient pathways and the evaluation of process differences that vary across healthcare provider institutions. Distinction could then be made between the delivery of care (e.g. treatments) and health service process characteristics (e.g. delays to treatment) that make up the total patient experience. The proposed methodology paves the way for a more advanced modelling approach to the analysis of treatment centre characteristics (in addition to patient casemix characteristics), where differences in the patient pathway of care are modelled to evaluate organisational features in relation to patient outcomes. Such strategies permit hypothesis generation around which healthcare delivery and organisational features warrant intervention, informing prospective cluster-randomised trials targeted at improving service organisation and delivery. This feeds into existing approaches for quality improvement research, consistent with the principles of the MRC framework for the development and evaluation of complex interventions [28].

## Conclusions

The main advantages of the MLLC approach are that it provides accurately derived estimates of the outcome differences across Trust classes, hence improved 'casemix adjustment' for individual Trusts. Trust level covariates may be included, capturing additional casemix complexity. Although deliberately simplified, our illustration demonstrates a principle that could readily extend to a number of more sophisticated scenarios (e.g. time-to-event analysis, multiple treatment centres, cross-classified structures). The MLLC model paves the way to adjust for variations in the patient pathway (especially delivery of appropriate healthcare), permitting the evaluation of institutional processes, which should provide a more robust approach to evaluating institutional performance than is current practice.

## Competing interests

The authors declare that they have no competing interests.

## Authors' contributions

MSG conceived the idea and planned the study, he drafted the manuscript, and coordinated input from all coauthors; WJH did the analysis, addressing the statistical problems throughout the course of analytical developments, she produced the results (tables and chart) and drafted the manuscript; AD contributed her expertise from previous work that led on to this study and commented on the manuscript; DF provided cancer epidemiology expert advice and commented on the manuscript; RMW contributed to initial discussions surrounding concept and study design, helped steer the analyses, and contributed to the interpretation of the results and the writing of the manuscript. All authors read and approved the final manuscript.

## Appendix

The multilevel latent class model used in this study takes the form:

where *y*_*ij *_is the outcome (*death *= 1, *alive *= 0) for patient *i *within Trust *j*;  is the vector of patient-level covariates; *t *are the Trust classes (1...*T*); and *c *are the patient classes (1...*C*); *p*(*c*|*t*) is the probability of being in patient class *c *conditional on being in Trust class *t*, and in this study *C *is taken as the same for each Trust. The patient class model, *P*^(^^*c*^^)^, expands to:

where *β*_0_^(^^*c*^^) ^to *β*_5_^(^^*c*^^) ^are the patient-class specific coefficients for the patient-level covariates.

## Pre-publication history

The pre-publication history for this paper can be accessed here:

http://www.biomedcentral.com/1472-6963/11/53/prepub
